# Haemodiafiltration elicits less platelet activation compared to haemodialysis

**DOI:** 10.1186/s12882-016-0364-x

**Published:** 2016-10-13

**Authors:** Gergely Becs, Renáta Hudák, Zsolt Fejes, Ildikó Beke Debreceni, Harjit Pal Bhattoa, József Balla, János Kappelmayer

**Affiliations:** 1 Department of Nephrology, University of Debrecen, Debrecen, Hungary; 2 Department of Laboratory Medicine, University of Debrecen, Nagyerdei krt. 98, Debrecen, Hungary; 3 MTA-DE Vascular Biology, Thrombosis and Hemostasis Research Group, Hungarian Academy of Sciences, Debrecen, Hungary

**Keywords:** ESRD, Haemodiafiltration, Haemodialysis, Monocyte-platelet aggregate, P-selectin

## Abstract

**Background:**

Mortality in patients with end-stage renal disorders is often a consequence of cardiovascular complications. Renal replacement therapies may contribute to this morbidity by promoting cellular activation. In renal failure patients peripheral blood samples were investigated for platelet and endothelial cell activation markers to compare the effects of haemodiafiltration (HDF) and haemodialysis (HD).

**Methods:**

Overall 28 patients were included in the study. Platelet P-selectin and leukocyte - platelet heterotypic aggregates were studied by flow cytometry. Soluble P- and E-selectin values were determined by ELISA, while von Willebrand factor (vWF) antigen levels were measured by immunoturbidimetry. Statistical analysis was done by the SPSS v22 software.

**Results:**

Platelet surface P-selectin was below 3.0 % in healthy controls, but it was higher during the dialysis after 4 h, 8 % and 14.3 % in HDF and HD, respectively. Monocyte-platelet heterotypic aggregates were significantly elevated after 4 h in both treatments, up to 69.2 % in HDF and to 82.9 % in HD. Soluble P-selectin levels were also significantly elevated by the end of both treatment procedures (*p* < 0.001), vWF antigen values, however, showed elevation only during HD treatment.

**Conclusions:**

The attenuated platelet activating effects of HDF compared to HD may contribute to a less unfavourable vascular effect in this treatment modality.

**Electronic supplementary material:**

The online version of this article (doi:10.1186/s12882-016-0364-x) contains supplementary material, which is available to authorized users.

## Background

Patients with end-stage renal disease (ESRD) undergo renal replacement therapy to avoid fatal complications of uremic toxins, elevated potassium level and overhydration. Renal replacement therapy involves mostly haemodialysis and in smaller proportion peritoneal dialysis treatments. Recent technological advancements have significantly contributed to improved efficacy of dialysis treatments. In the clearance of waste products, the basic modality is haemodialysis, where blood is cleared by the counter-flow of the dialysis fluid by the effect of diffusion. Despite advancements in ESRD treatment, the 3-year mortality in this patient group is 50 %, which is primarily contributed to cardiovascular complications [1].

Haemodiafiltration (HDF) was introduced in the mid seventies and was developed to increase the clearance of substances with middle molecular weight and the physical background of this modality is the convective transport. It is widely used in Europe, with limited use in Japan or Australia and is not approved in the United States, mostly due to concerns of appropriate water quality, since in this treatment the substitution fluid is reinfused into the patient [2]. Nevertheless, several studies have reported improved survival of patients on HDF versus HD in recent years, if substitution fluid volume is higher than 18 L per session [3–5].

Numerous authors have addressed the question of coagulation and platelet activation in haemodialysed patients. One important finding is that cardiovascular events were linked to the tissue factor (TF) system [6] and that in ESRD patients an elevated level of monocyte expressed TF along with other adhesion molecules like CD11b/18 were reported. This was even more pronounced in ESRD patients under haemodialysis [7]. Both of these receptors contribute considerably to the procoagulant potential of blood. In previous studies dealing with extracorporeal circulation, we also identified significant leukocyte activation by investigating the same receptors involved in blood coagulation initiation [8, 9]. Several studies have dealt with platelet activation in HD patients describing aspects of platelet count and function [10, 11] as well as prothrombotic changes in platelet, endothelial and coagulation systems [12], but only one paper addressed the question of platelet activation in HD versus HDF and the results were published as a side study of the CONTRAST trial [13]. These authors described enhanced platelet-associated activation markers in HDF patients, but decreased degranulation of platelets as measured by the soluble marker beta-thromboglobulin [13], consequently the cellular and soluble marker results were somewhat contradictory.

Here, we investigated - in the same set of ESRD patients - the effect of two different renal replacement therapies on platelet and endothelial activation, by investigating cell-associated and soluble markers.

## Methods

### Patient enrolment and dialysis procedures

Twenty-eight patients with ESRD were selected for an interventional longitudinal non-randomized study, each subject qualified as its own control. Exclusion criteria included diabetes mellitus, as this condition may cause platelet activation. Similarly patients with cancer, haematological or haemostasis disorders were excluded. Furthermore, any patient on anti-platelet drugs was also excluded. The Ethics Committee of the University of Debrecen approved the study protocol. The patients – after receiving detailed information about the trial – gave written informed consent. The age of participants was between 18 and 70 (13 males, 15 females, mean age 53 ± 13.8 years), and each patient was on dialysis for at least 3 months before the study was started (mean length 89.5 ± 74.5 months).

The patients underwent dialysis three times a week employing Fresenius 5008S devices (Fresenius Medical Care, Bad Homburg, Germany), and one session lasted for 250 min (to reach the expected efficiency in 5 cases the treatment lasted for 270 min, but the blood sampling also took place after 4 h). Cordiax FX dialysers, FX60, FX600 and FX800 respectively were used (Fresenius) for the treatments, which were matched to patients weight and blood flow rate as the effectiveness required it. Effectiveness was calculated during each treatment employing Online Clearance Monitoring Kt/V (OCM Kt/V; Fresenius Medical Care, Bad Homburg, Germany) measurements. OCM Kt/V should be higher, than 1.3 in hemodialysis and more than 1.4 during haemodiafiltration. Haemodiafiltration was performed with the average Kt/V of 1.67 ± 0.21, while only 1 patient could not reach the target. In hemodialysis the Kt/V value was 1.48 ± 0.2 on average and 3 patients were under 1.3. In most cases the FX60 with 1.4 m^2^ was optimal (24 patient). In order to provide efficient dialysis we employed FX600 for 3 patients with dialyser surface of 1.6 m^2^ or FX800 for 1 patient with 2.0 m^2^. The type of dialysers was not changed during the study between the modalities. Cordiax FX membranes represent the latest Helixone®plus membranes with INLINE steam sterilization assuring excellent biocompatibility. The same speed of blood flow (mean 366.1 ± 63.7 mL per minute) and dialysate flow (439.3 ± 76.5 mL per minute) was used in both modalities. In order to remove the excess fluid the net ultrafiltration during haemodialysis and haemodiafiltration was 2703.6 ± 1118.0 mL per session and 2432.1 ± 1014.4 mL per session, respectively. At our dialysis centre we use HDF as the standard modality, so in the beginning of our study we started the sampling with this method. To avoid any disturbing factor we waited for 2 weeks after switching to HD before measuring the platelet markers in HD treatment. Avoiding any influence of anticoagulation we used the same amount of unfractionated heparin during both types of treatments. The heparin dose was adjusted to the patient’s need to avoid blood line coagulation and bleeding from AV-fistules. In all cases heparinization was stopped 30 min before the end of the session. During haemodiafiltration the volume of substitution fluid was 23.4 ± 3.8 l. The substitution fluid was prepared on-line from dialysis solution through a set of two membranes to purify it before direct infusion into the blood line. The substitution fluid was manufactured on-line from ultrapure water and consisted of 138 mmol/L sodium, 2 or 3 mmol/L potassium depending on the patient’s potassium value, 1.25 mmol/L calcium, 0.5 mmol/L magnesium, and 1 g/L glucose. A two-week adjustment period was chosen before the treatment modality was switched for HD and the samples were collected from the same patients again. The bicarbonate dialysis solution contained the same amount of solutes as described above. Bicarbonate concentration of dialysate/replacement fluid was adjusted between 28–38 mmol/L to obtain plasma bicarbonate level of 20–22 mmol/L prior to dialysis. The purity and sterility of dialysis and substitution fluids met the criteria of the 8^th^ edition of European Pharmacopoeia (endotoxin level <0.01 EU/mL, bacterial counts <0.1 CFU/mL and any heavy metal ions <0.01 mg/L).

### Blood collection and cell counting

Blood samples were taken from the efferent line port into sodium citrated tubes (3.2 %, BD Vacutainer) at three time points, at 4 min, 1 and 4 h after the onset of dialysis. In contrast to see the exact activation in the beginning we dedicatedly took 0 min samples from arterial needles and 4 min samples from efferent line only in ongoing HDF treatments. Cell counting was carried out by the Advia 2120 Haematology Analyzer (Siemens, Dublin, Ireland) within two hours of blood collection and cell numbers were adjusted for the haemoconcentration by using the red blood cell counts.

### Platelet poor plasma (PPP) preparation and measurement of soluble markers

Whole blood was centrifuged at 1500 x g for 15 min at room temperature, albumin levels were immediately measured by a Cobas C8000 Clinical Analyzer (Roche Diagnostics, USA). Soluble P-selectin and E-selectin levels were measured by a commercial ELISA kit (R&D Systems, Minneapolis, MN, USA) according to the manufacturer’s instructions, while von Willebrand Factor (vWF) antigen levels were measured by turbidimetry (BCS XP, Siemens, Germany). Levels of soluble proteins were adjusted for the haemoconcentration by normalizing the results for albumin values.

In randomly selected patients who underwent HDF, heparin content of plasma was measured based on the activated factor X (anti-Xa) inhibitory assay (Berichrom® Heparin, Siemens). This is a chromogenic assay for the determination of the activity of heparin (conventional or low-molecular-weight) in plasma. Factor Xa is inactivated by antithrombin III (AT III) during the incubation phase of the test and the reaction is catalyzed by heparin. The quantity of residual FXa is determined via the increase in absorbance at 405 nm, using a chromogenic substrate in a kinetic test.

### Flow cytometric assays

The expression of platelet surface P-selectin and leukocyte - platelet heterotypic aggregates were measured by flow cytometry (FC500 flow cytometer, Beckman Coulter). To standardize measurement conditions and to minimize in vitro platelet activation whole blood was fixed for P-selectin measurement and labelled for heterotypic aggregate determination within 2 h after blood collection. After fixation and washing by PBS, samples were labelled by CD42a-FITC and CD62-PE (Becton Dickinson Biosciences) for 20 min, washed twice and analyzed. Whole blood samples were incubated with CD14-PE and CD42a-FITC for 15 min to detect leukocyte-platelet heterotypic aggregates. Results were always compared to samples stained with non-immune IgG that served as isotype controls. Red blood cells were lysed and the samples were washed twice and fixed and subsequently analyzed.

### Statistical analysis

Kolmogorov-Smirnov test was used for the evaluation of the normality of the data. Most parameters were non-normally distributed; univariate analyses were performed by Mann–Whitney U-test or the independent samples t-test. For paired analyses, as required for the related samples, Wilcoxon signed ranks test or the paired-samples t-test were performed. The Spearman’s ρ value was calculated for correlation analysis. All analyses were performed using the SPSS Statistics software, version 22.0 (IBM Corps., Armonk, NY, USA).

## Results

### Evaluation of the changes during dialysis

Both dialysis procedures cause a considerable fluid loss, thus there is a variable degree of haemoconcentration that was monitored by measuring the red blood cell (RBC) count and albumin values at each sampling time. The increase in RBC counts and albumin were significant between sampling times (Additional file 1: Figure S1), by using both treatment modalities. Thus, cell counts were normalized for RBC and soluble markers were normalized for albumin values. Platelet counts - adjusted for the haemoconcentration - significantly decreased during the four-hour observation period that suggests a considerable sequestration of these cells, most likely on the dialyzer surface (Additional file 2: Figure S2).

### Direct platelet activation markers

**Fig. 1:**
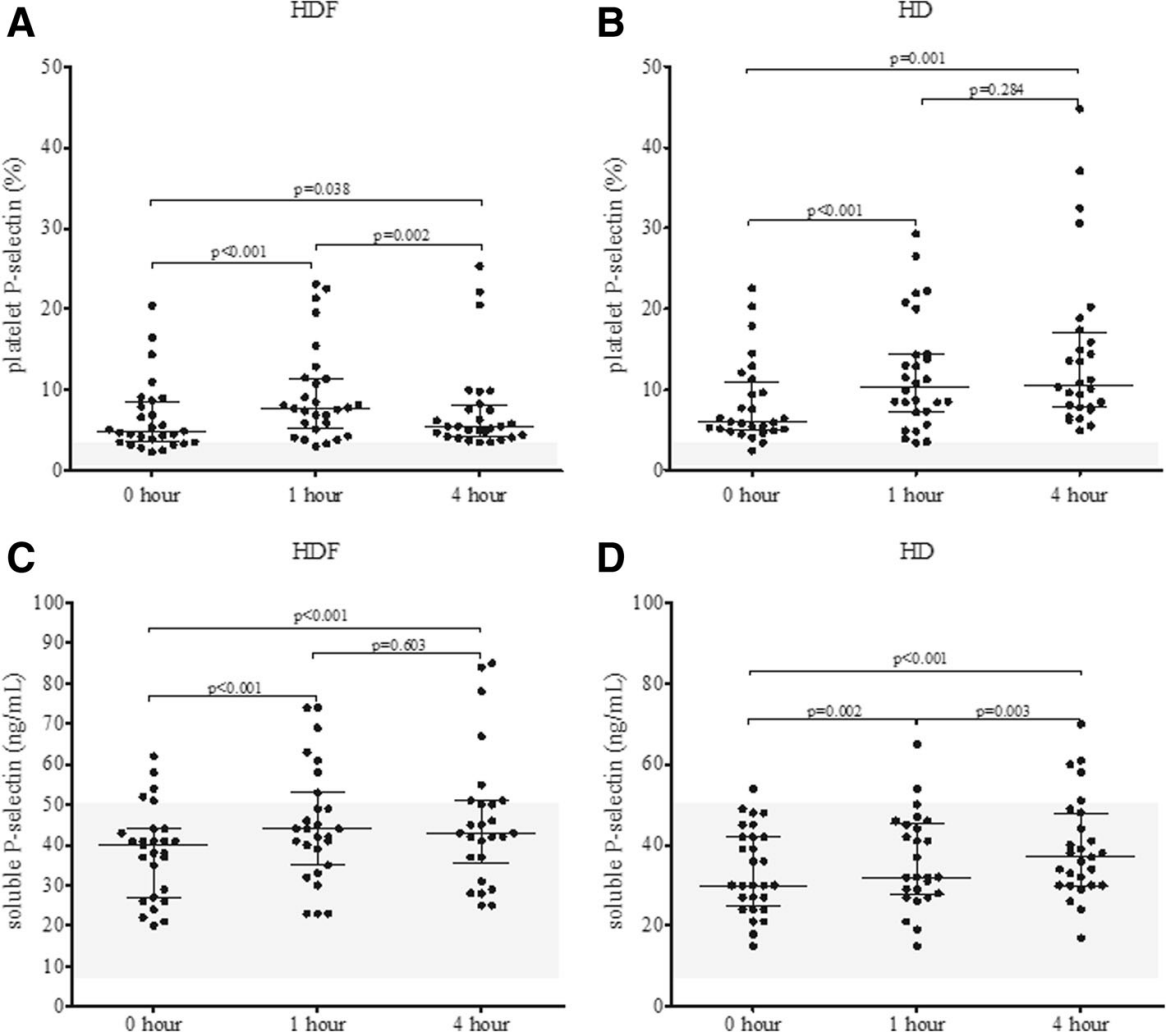
Changes in platelet P-selectin (**a** and **b**) and soluble P-selectin (**c** and **d**) during haemodiafiltration (HDF) and haemodialysis (HD) at 0, 1 and 4 h. The shaded area represents the reference range for the respective parameter on this and subsequent figures. *P* values were calculated by Wilcoxon signed ranks test

Platelet P-selectin values were well over the reference range of < 3 %, as described earlier [14], right from the beginning of the dialysis procedures (Fig. 1 panels a, b). It needs to be emphasized that the treatment requires the dialyzer to be filled up with heparinized blood, and this is achieved by a 4–5 min roller pump-assisted extracorporeal circulation. As such, a few minutes have already elapsed when the initial sample (0 h) was collected. HD treatment resulted in higher platelet P-selectin values when compared to the HDF procedure and the difference was the largest by the end of the procedure (Table 1). Although the median soluble P-selectin (sP-selectin) values were within the reference range by the end of both procedures, several results exceeded the upper reference limit (Fig. 1 panels c, d). The sP-selectin values increased during the procedure, but significant differences were observed between the two treatment modalities at the 1 h time-point, with the values being higher during HDF (Table 1).

**Table 1:** *P* values upon comparing the studied parameters at 0, 1 and 4 hours during haemodiafiltration (HDF) and haemodialysis (HD). n.s.: *p* > 0.05

Parameters	0 hour			1 hour			4 hour		
	HD (mean ± SEM)	HDF (mean ± SEM)	*P* value	HD (mean ± SEM)	HDF (mean ± SEM)	*P* value	HD (mean ± SEM)	HDF (mean ± SEM)	*P* value
Platelet P-selectin (%)	8.26 ± 0.98	6.5 ± 0.84	n.s.	12.00 ± 1.33	9.50 ± 1.11	n.s.	14.45 ± 1.92	7.56 ± 1.07	*p* < 0.001
Soluble P-selectin (ng/mL)	33.68 ± 1.99	38.26 ± 2.17	n.s.	36.08 ± 2.28	45.07 ± 2.75	*p* = 0.022	39.22 ± 2.40	46.15 ± 3.26	n.s.
Monocyte-platelet aggregate (%)	64.82 ± 3.57	47.61 ± 2.57	*p* < 0.001	76.79 ± 3.06	65.82 ± 3.28	*p* = 0.010	83.18 ± 2.52	67.79 ± 3.48	*p* = 0.001
Neutrophil-platelet aggregate (%)	13.75 ± 1.73	9.54 ± 0.84	*p* = 0.024	17.96 ± 1.81	15.29 ± 1.62	n.s.	24.46 ± 2.57	19.50 ± 2.92	*p* = 0.032
Soluble E-selectin (ng/mL)	24.75 ± 2.42	25.58 ± 2.14	n.s.	25.08 ± 2.75	26.46 ± 2.09	n.s.	24.37 ± 2.24	25.54 ± 2.07	n.s.
vWF antigen (%)	158.60 ± 10.25	158.30 ± 9.31	n.s.	161.40 ± 10.82	166.10 ± 9.93	n.s.	169.80 ± 10.69	162.80 ± 10.66	n.s.

*P* values were calculated by Wilcoxon signed ranks test

### Platelet-leukocyte aggregates

**Fig. 2:**
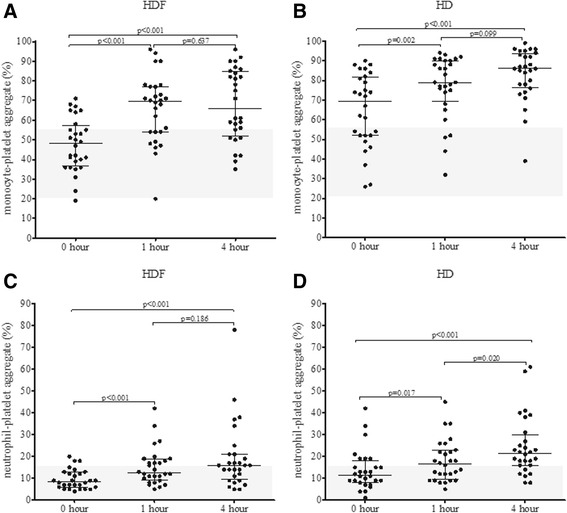
Changes in monocyte-platelet aggregates (**a** and **b**) and neutrophil-platelet aggregates (**c** and **d**) during haemodiafiltration (HDF) and haemodialysis (HD) at 0, 1 and 4 h. Wilcoxon signed ranks test was employed to calculate *p* values

Platelet activation was also monitored by using the indirect platelet activation tests where we examined heterotypic cell aggregates. Here, a striking elevation was observed when monocyte-platelet aggregates were analyzed compared to a mean value of 38 % obtained in controls and previously published by us and others [15, 16]. The ratio of these aggregates kept increasing during the dialysis and the number of these aggregates were significantly higher in HD treatment at each analysis time point (Fig. 2a, b) and a similar tendency could be observed in case of neutrophil-platelet aggregate ratio during dialysis (Fig. 2c, d). At each sampling time point HD treatment resulted in significantly higher monocyte-platelet aggregate formation than HDF treatment.

### Control measurements

**Fig. 3:**
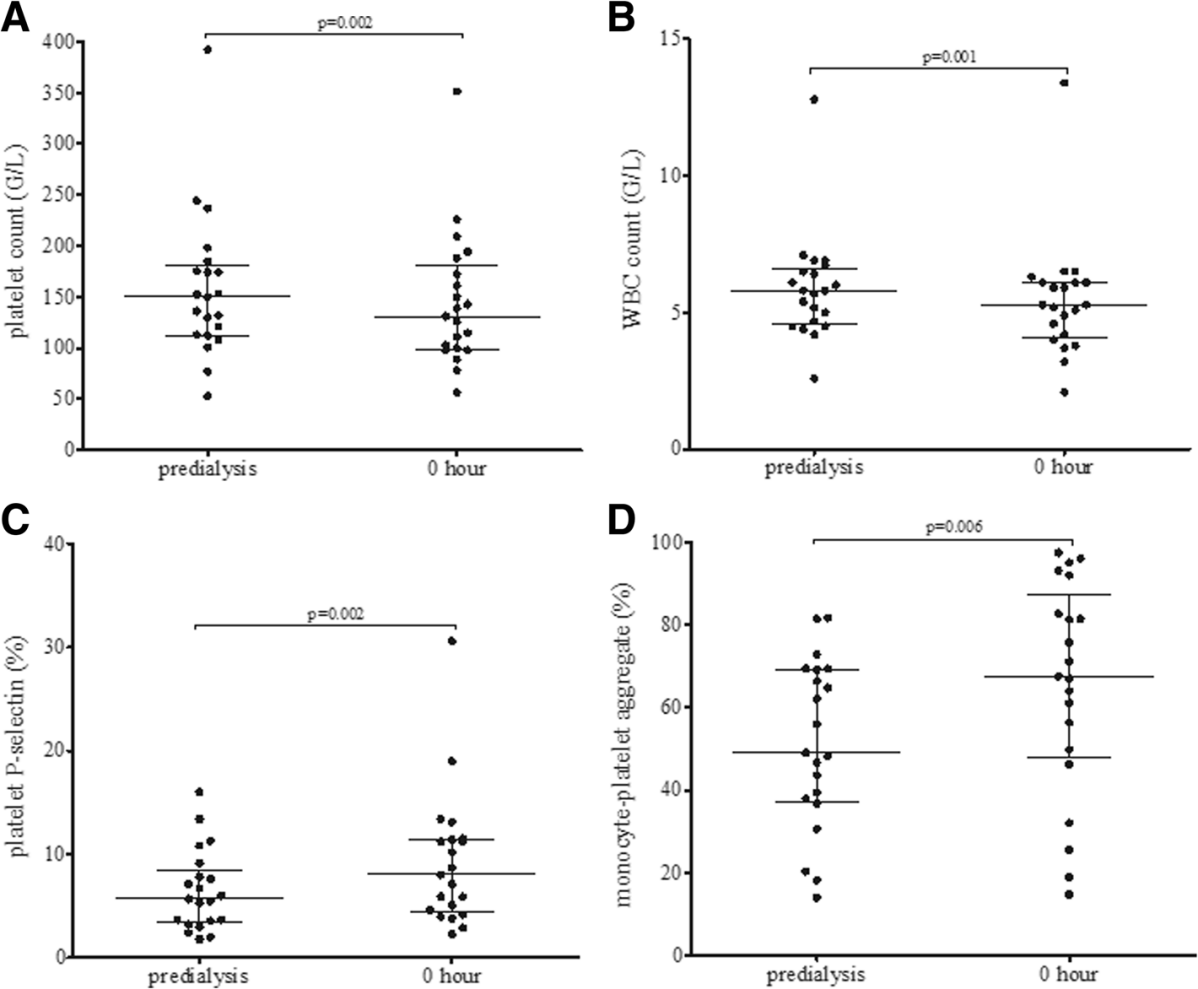
Changes in platelet count (**a**), white blood cell count (WBC) (**b**), platelet P-selectin (**c**) and monocyte-platelet aggregates (**d**) during haemodiafiltration (HDF) at predialysis and 0 h. *P* values were calculated by Wilcoxon signed ranks test

Since the above elevations were significant, we set out to determine the effect of potential variables on the studied direct and indirect platelet activation markers. First, we investigated the effect of blood flow rate. At 0 h, in the HD and the HDF groups, blood flow rate did not correlate with the studied platelet activation markers (surface P-selectin, soluble P-selectin, monocyte, and neutrophil heterotypic aggregate). In the HD and the HDF groups, the blood flow rate also did not correlate with the percentage change between the 0 and 4 h values of the studied platelet activation markers, thus we can conclude, that the observed alterations are not related to blood flow rate. Furthermore, platelet activation markers showed no correlation with the duration of treatment months of renal replacement therapy. Subsequently, we introduced another set of measurements where we hypothesized that the high platelet activation markers observed already in the 0 h samples could be due to platelet and/or leukocyte activation during the initial minutes when the dialysis system was filled up (these were the predialysis samples). Indeed in this series of measurements, we observed that there was a significant decrease in platelet and leukocyte counts between predialysis and 0 h samples (Fig. 3a, b), furthermore, platelet activation markers were also increased in the 0 h samples compared to the predialysis samples (Fig. 3c, d).

Since heparin may also activate platelets, we thought to examine whether the observed changes in platelet activation markers could be elicited by the heparin utilized during these treatments. To determine the effects of heparin on platelet activation markers in ESRD patients during dialysis, we conducted an experiment in which we added in vitro unfractionated heparin (UFH) to the predialysis samples from 3 ESRD patients in a range of heparin concentrations as determined by the anti-Xa assay. Samples were incubated with 0.5 U/mL or 2.0 U/mL UFH for 5 or 60 min. The surface expression of P-selectin and the leukocyte-platelet heterotypic aggregates were measured by flow cytometry and the results were compared to non heparinized samples. In vitro heparinization did not augment the level of the activation marker of platelets neither the amount of heterotypic aggregates (Additional file 3: Figure S3).

### Endothelial cell activation

**Fig. 4:**
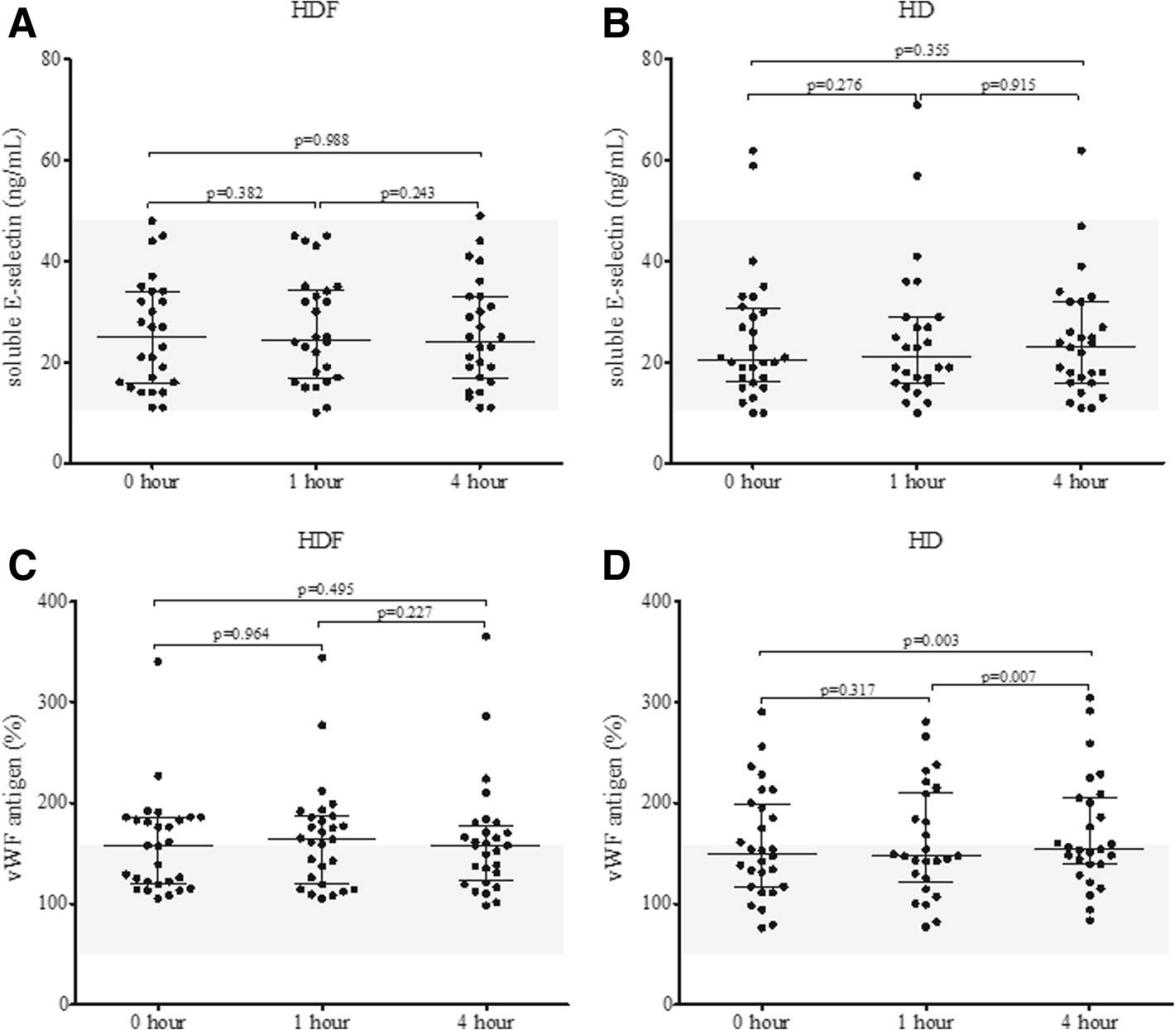
Changes in soluble E-selectin (**a** and **b**) and von Willebrand Factor (vWF) antigen (**c** and **d**) during haemodiafiltration (HDF) and haemodialysis (HD) at 0, 1 and 4 h. Wilcoxon signed ranks test was employed to calculate *p* values

Since endothelial injury has also been described during extracorporeal circulation we measured two endothelium associated biomarkers the soluble E-selectin (sE-selectin) and the vWF antigen. At each sampling time, there were only few sE-selectin values above the manufacturer suggested upper reference limit (51 ng/mL), however the median sE-selectin was not elevated during the procedures and there were no difference between treatment modalities. Also, no difference was observed between dialysis procedures for vWF antigen, in contrast to sE-selectin but vWF antigen values were elevated (>150 %) right from the beginning and significantly increased by the end of the HD procedure (Fig. 4).

## Discussion and conclusions

In summary of our present study we found novel changes in platelet activation markers during the two different dialysis treatment modalities. Platelet and soluble P-selectin values demonstrated increasing levels during the treatments, but there were a few significant differences when the two modalities were compared. Heterotypic aggregate formation was also elevated during both modalities especially in HD. Monocyte-platelet aggregates showed significantly higher levels during HD compared to HDF. Changes in neutrophil-platelet aggregates showed increasing levels in a time-dependent manner but with only lower significancy levels. Endothelial activation markers, such as E-selectin and von Willebrand factor, were observed in HD group and only vWF elevation was significant during the treatments.

Individual data from our work according to previous studies and literature can make a more complex view of haemostatic abnormalities in chronic kidney diseases, since in this patient group both bleeding and thrombotic complications are observed and this dual effect is also characteristic for platelets. In CKD patients, platelet dysfunction is frequently observed that may be caused by the uremic toxins, enhanced nitric oxide production and anaemia, but the platelets of dialyzed patients are constantly activated and these repeated procedures result in the ’exhausted platelet syndrome’ [17, 18]. Even in early reports it was described, that platelet-leukocyte aggregates occur during dialysis, most likely related to the primary platelet activation [19]. Already at this time the authors suggested this test as a biocompatibility marker. Further studies identified platelet trapping, particularly sequestration of the larger platelets in the circuit, and suggested that soluble markers like beta-thromboglobulin are suitable for detecting platelet degranulation [20]. Another methodological approach to investigate platelet activation was published by Schoorl and colleagues, who verified platelet degranulation by light and electron microscopy [21]. Nevertheless, in more recent reports the biocompatibility of the dialyzer membranes was monitored by the platelet count and it was concluded, that the more biocompatible newer membranes like the polysulfones do not result in marked thrombocytopenia [22]. In our studies we found by using a highly biocompatible membrane, that the platelet count did not change dramatically however, a significant platelet count decrease was observed with both treatment modalities.

In addition to platelet activation, leukocyte activation may occur during HD and contribute to heterotypic aggregate formation. CKD patients are exposed persistently to a low-grade inflammation. The level of inflammatory markers is affected by numerous factors such as transient infections, several comorbidities, and the intermittent stimulus of the dialysis procedure [23]. The archetype inflammatory marker, C-reactive protein, has long been shown to be able to elicit procoagulant functions by stimulating monocyte tissue factor expression [24]. Furthermore, the binding of activated platelets, to monocytes and neutrophils, via P-selectin require P-selectin glycoprotein ligand 1 (PSGL-1) that has been shown to be redistributed by activated cytokines [25].

We have identified that flow cytometry based assays are the most sensitive in identifying platelet and/or leukocyte activation, when comparing the two different treatment modalities. The considerable elevation in the gold-standard platelet P-selectin suggests that the very high heterotypic aggregate rate between the myeloid cells and platelets are largely due to primary platelet activation as proposed recently [26]. This phenomenon occurs very early during dialysis as significant elevations could be observed after a few minutes of system filling compared to baseline values. Since at this early time point there is minimal recirculation this may have been caused mostly by the enhanced turbulence. In previous clinical conditions we also found augmentation compared to controls in platelet P-selectin and the monocyte-platelet ratio in obese and diabetic patients and during coronary stenting [14, 15, 27]. However, the degree of the elevation of these aggregates in such patient groups were well below that observed during our present study on HD and HDF treatments. Although a previous study has postulated a role for these aggregates in the pathophysiology of vascular disorders e.g., the development of aortic stiffness, the pathomechanism is still to be elucidated [28]. Nonetheless, soluble factors are likely to play a role in mediating platelet/leukocyte activation, since their effect may be propagated via the plasma, probably by activating complement factors [29].

We previously identified that haemodiafiltration was found to have beneficial effect on echocardiographic markers representing left ventricular diastolic function [30]. We investigated the changes in platelet activation markers to evaluate the effect of different types of dialysis treatments. In this present study on the comparison of the two treatment modalities we also observed beneficial effects on direct and indirect platelet activation markers of HDF compared to HD. The dialysis was performed using dialysers with different effective surface area (FX60: 1.4 m^2^; FX600: 1.6 m^2^; FX800: 2.0 m^2^, adjusted to the patient’s weight, dialysis efficiency, etc.) and acknowledging the limitation of statistical analyses with small sample sizes (FX60: 24 patients, FX600: 3 patients and FX 800: 1 patient), which in the present case are way below the limit of safe statistical handling, we found no statistically significant differences comparing the 3 membrane surface groups.

Although in a previous meta-analysis a similar sensitivity could be observed in vascular disorders for soluble and platelet-associated P-selectin [31] the simultaneous investigation of these markers during dialysis treatments showed that platelet P-selectin is having a superior sensitivity compared to soluble P-selectin. As was already suggested by Michelson [32] probably the most sensitive platelet activation assay is the measurement of the monocyte-platelet conjugates, as platelets may lose P-selectin in the circulation. In our patient cohort, we found that such platelet-leukocyte aggregates also had an excellent sensitivity as it showed nearly maximal values that can usually be observed only in agonist stimulated samples during in vitro experiments. In contrast to platelet activation markers, the soluble endothelial cell activation marker E-selectin did not show any change in kinetics. E-selectin is synthesized de novo in stimulated endothelial cells and its expression and release requires considerable time. Unlike E-selectin, vWF is stored in the endothelial Weibel-Palade bodies and can readily be mobilized when the endothelial cells are activated. It is plausible that the elevated levels experienced during the renal replacement therapies are a result of this difference and most likely this is why in HD, vWF antigen increased significantly by the end of the procedure. It should also be noted that the Weibel-Palade bodies of activated endothelial cells can also release soluble P-selectin, however even with this potential contribution, the sensitivity of this parameter was below that of the cell associated markers. The molecular weights of measured markers of platelet activation, such as E-selectin, P-selectin and vWF antigen are 115 kDa, 140 kDa and 250 kDa (monomer), respectively. The diameter of monocyte-platelet aggregates is at least 15 μM. The Sieving coefficient of FX Cordiax® dialysers for albumin is less, than 0.001 according to the manufacturer’s description. Thus, these large molecular weight substances are unable to cross the capillary membrane in the dialysers.

Based on these observations, we propose that the more gentle effects of HDF compared to HD may contribute to a less unfavourable vascular effect in this treatment modality. The underlying mechanism can be explained by the fundamental physical processes during dialysis treatment methods. Although both modality use the same type of dialyser, with the same pore-size, while HD employs only diffusion for excretion HDF is applying convective transport as well. Convective transport alone can increase dramatically the excretion of small to mid-size proteins with the molecular weight 5–40 kDa and its also increases the excretion of smaller molecules compared to HD. Further studies are required to determine whether the decreased platelet activation can be explained by the increased elimination of the enhancers of platelet activation during HDF.

## Additional files

Additional file 1: Figure S1. Red blood cell count (panels A, B) and albumin values (panels C and D) constantly increase during both treatment modalities as a result of haemoconcentration. Paired-samples t-test was employed for red blood cell count and Wilcoxon signed ranks test for albumin to calculate *p* values. (TIF 104 kb)
Additional file 2: Figure S2. Platelet count is decreased as they are sequestered during both procedures. Paired-samples t-test was employed to calculate *p* values. (TIF 105 kb)
Additional file 3: Figure S3. No significant changes (n.s.) were observed in platelet P-selectin (A), monocyte-platelet aggregates (B) and neutrophil-platelet aggregates (C) at different concentrations of unfractionated heparin (UFH) following 5 and 60 min of incubation. *P* values were calculated by Wilcoxon signed ranks test. (TIF 189 kb)
Additional file 4: Raw data of the study. (XLSX 32 kb)

## Abbreviations

AT III: Antithrombin III
ESRD: End-stage renal disease
HD: Haemodialysis
HDF: Haemodiafiltration
PPP: Platelet poor plasma
PSGL-1: P-selectin glycoprotein ligand 1
RBC: Red blood cell
sE-selectin: Soluble E-selectin
sP-selectin: Soluble P-selectin
TF: Tissue factor
UFH: unfractionated heparin
vWF: von Willebrand factor
