# Supplementary figures and images for: Haemodiafiltration elicits less platelet activation compared to haemodialysis
Source: BMC Nephrol. 2016 Oct 13;17:147. doi: 10.1186/s12882-016-0364-x (PMC5064778; doi:10.1186/s12882-016-0364-x)

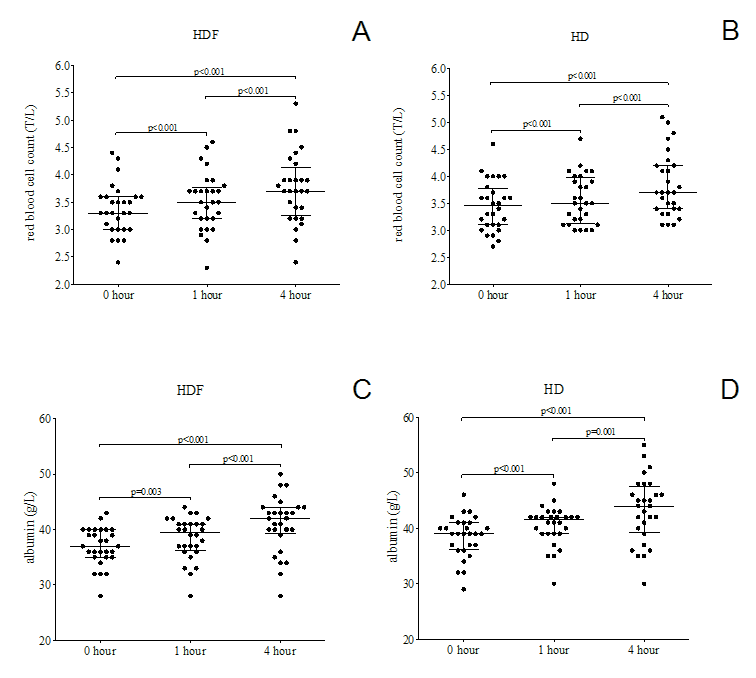

Supplement: Additional file 1: Figure S1. — Red blood cell count (panels A, B) and albumin values (panels C and D) constantly increase during both treatment modalities as a result of haemoconcentration. Paired-samples t-test was employed for red blood cell count and Wilcoxon signed ranks test for albumin to calculate p values. (TIF 104 kb) [file 12882_2016_364_MOESM1_ESM.tif]

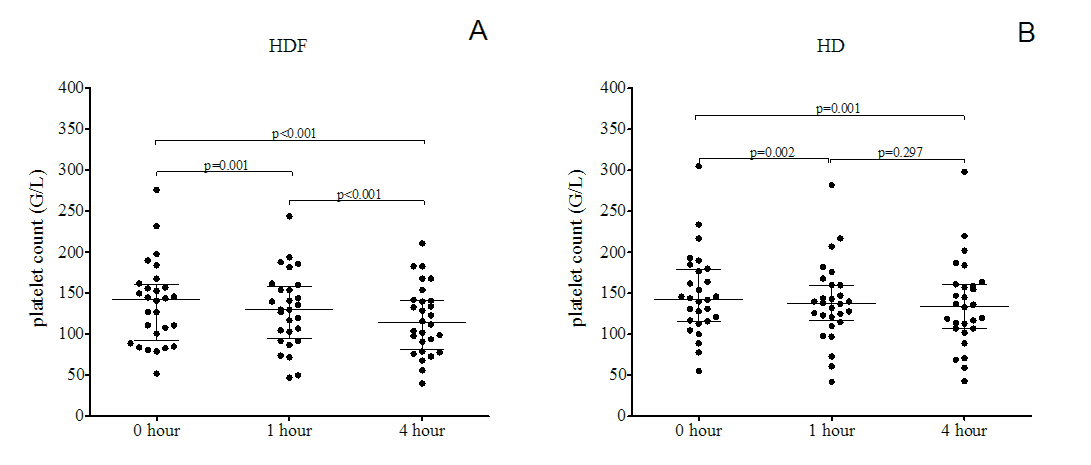

Supplement: Additional file 2: Figure S2. — Platelet count is decreased as they are sequestered during both procedures. Paired-samples t-test was employed to calculate p values. (TIF 105 kb) [file 12882_2016_364_MOESM2_ESM.tif]

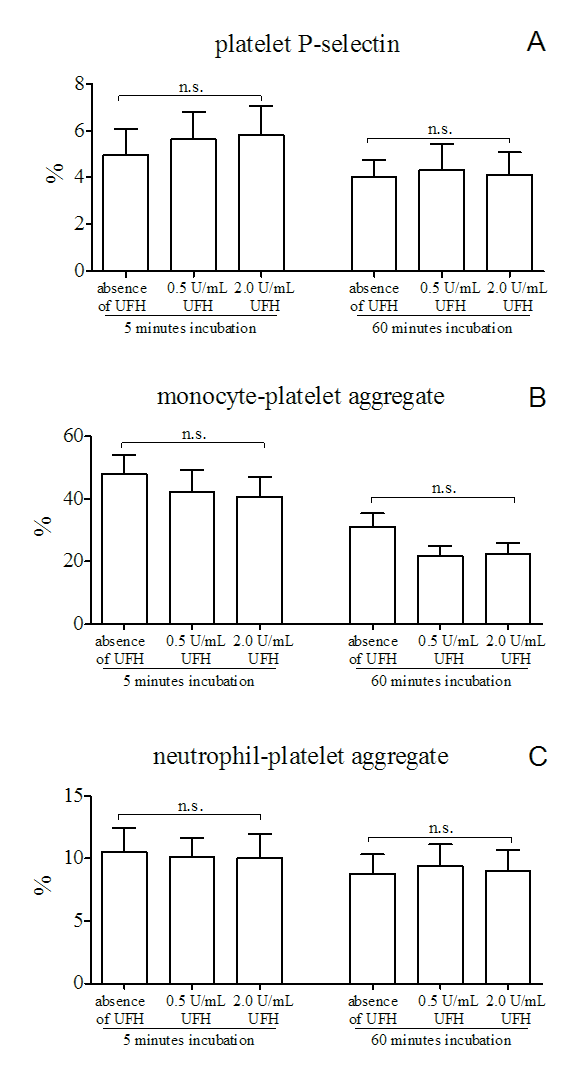

Supplement: Additional file 3: Figure S3. — No significant changes (n.s.) were observed in platelet P-selectin (A), monocyte-platelet aggregates (B) and neutrophil-platelet aggregates (C) at different concentrations of unfractionated heparin (UFH) following 5 and 60 min of incubation. P values were calculated by Wilcoxon signed ranks test. (TIF 189 kb) [file 12882_2016_364_MOESM3_ESM.tif]
